# The comprehension and production of Wh- questions among Malay children with developmental language disorders: Climbing the syntactic tree

**DOI:** 10.3389/fpsyg.2022.948992

**Published:** 2022-10-28

**Authors:** Norsofiah Abu Bakar, Giuditta Smith, Rogayah A. Razak, Maria Garraffa

**Affiliations:** ^1^School of Humanities, Universiti Sains Malaysia, Pulau Pinang, Malaysia; ^2^Faculty of Medicine and Health Sciences, University of East Anglia, Norwich, United Kingdom; ^3^Department of Education, Faculty of Social Science & Liberal Arts, UCSI University, Kuala Lumpur, Malaysia

**Keywords:** Malay, DLD, Wh- questions, clinical markers, which-questions, subject/object asymmetry

## Abstract

This study is an investigation of both comprehension and production of Wh- questions in Malay-speaking children with a developmental language disorder (DLD). A total of 15 Malay children with DLD (ages 7;0–9;11 years) were tested on a set of Wh- questions (*who* subject and object, *which* subject and object), comparing their performance with two control groups [15 age-matched typically developing (TD) children and 15 younger TD language-matched children]. Malay children with DLD showed a clear asymmetry in comprehension of Wh- questions, with a selective impairment for which NP questions compared with who questions. Age-matched controls performed at ceiling in all Wh- questions, while the language-matched group reported a subject/object asymmetry selective for the which NP, as reported in other languages. In production, both children with DLD and younger children showed a preference for questions with *in situ* Wh- elements, a structure that is allowed in colloquial Malay, but which is not produced by the age-matched TD group. Several non-adult-like strategies were adopted particularly by the children with DLD to avoid complex sentences, including substitution with yes/no echo questions, production of the wrong Wh- question, and use of a generic Wh- element. The study provides an insight on the mastery of Wh- questions in both typical Malay children and children with DLD. Implications for the definition of a clinical marker for DLD in a free word order language with Wh- *in situ* option will be discussed.

## Introduction

Children with developmental language disorder (DLD) can be defined as children with impairment in acquiring language components, often selective to a specific linguistic domain, such as syntax or phonology (Stark and Tallal, 1981; Leonard, 1998; Novogrodsky and Friedmann, 2009). As was proposed in the last consensus paper aiming at agreeing on the definition of DLD (Bishop et al., 2017), the language ability of children with DLD is not consistent with their age-group, and this is not attributed to factors external to the language system, such as hearing impairments, cognitive delays, or oral motor and neurological impairments, but rather to a specific impairment in the language system (Bishop, 2006, 2017). In the present paper, we present a study exploring the nature of the impairment in Malay children, particularly focusing on Wh-questions. The study was funded by the Research University Grant (GUP) (code UKM-GUP-2011-134) of the Universiti Kebangsaan Malaysia and the research grant PHUMANITI6315272 of the Universiti Sains Malaysia.

One of the issues in the study of the specific language profiles in children with DLD is that theories on language acquisition are often derived from considerations based on adult language and do not take into account the process of development, adopting a strict definition of correctness based on adult competence (Wexler, 1998, 2003). For example, in the domain of syntactic abilities, the debate on how grammar is acquired and which factors contribute to reaching grammatical competence is still ongoing in developmental linguistics, with few collaborations between linguists and developmental psychologists, indicating a need for more evidence across languages. Although the milestones of language acquisition have been uncovered to a good level of detail, thanks to crosslinguistic studies in different populations and the refinement of the underlying linguistic theory, it is still unclear whether there are interdependencies between acquisition of specific aspects of grammar and predicted stages of development and/or cognitive capacities ancillary to language (e.g., working memory or attention), with few studies tracking the development of a specific grammatical structure in both typical and atypical development (see Guasti, 2017 for an overview of the growth of grammar).

A phenomenon has been studied extensively in the acquisition of syntax is the acquisition of interrogative sentences (see Thornton, 2016 for an overview). Languages can vary on some specific syntactic properties, for example, allowing the presence of a Wh- element in its base position or fronting the element emphasizing its discourse relevance. For instance, the author reported non-adult-like productions of Wh- questions with referential which NP Wh- phrases in children aiming to avoid more complex configurations. Since Thornton’s seminal paper on production of Wh- in children (Thornton, 1995), more subtle distinctions have been reported in the acquisition of interrogatives.

In a relevant cross-sectional study, De Vincenzi et al. (1999) looked at the development of Wh- questions in 3- to 11-year-old children focusing in particular on the comprehension of which NP questions in Italian. 352 typically developing (TD) children were presented with *who* and *which* subject/object reversible questions in a thematic assignment picture selection task where children were asked to answer questions of the *who/which did what to whom?* kind. While comprehension of subject questions was above chance already in the youngest group, comprehension of reversible object questions appeared to be delayed. Furthermore, a clear distinction between *who* and *which* questions emerged, with the latter being more delayed and systematically lower in performance than who object questions across all age-groups. This asymmetry was used as proof to rule out the hypothesis that the main problem in the acquisition of Wh- sentences with non-canonical word order, such as object questions, was an overall delay in any sentence type with non-canonical word order. Rather, a more fine-grained distinction of the syntactic factors at play was needed. Similar results were reported with English-speaking children, who showed above chance performance on *who* questions (both subject and object) but lower accuracy in both production and comprehension of *which* questions, and in particular object *which* questions (Yoshinaga, 1996; Avrutin, 2000; Hirsch and Hartman, 2006). The overall picture for English-speaking children is a clear subject/object asymmetry in *which* questions also reported for Hebrew children at the age of 4 years (Friedmann et al., 2009). Finally, in a recent study on comprehension of *who/which* questions in a group of 47 English children (mean age 5;2 years), it was reported that *which* questions do not correlate with general grammatical knowledge measured with a standard grammatical test (Bishop Dorothy, 2003; Bishop, 2006). These results advocate for a distinct grammatical process for *which* questions that needs to be acquired and is independent from other grammatical processes (Riches and Garraffa, 2017).

To better explore the delay reported in many languages on both *who* and *which* object questions, a study was conducted to explore the role of similarity between the arguments during thematic role assignment (Guasti et al., 2012). In the task, the number features of the two nouns were mismatched in a transitive reversible sentence; if the agent was plural, the patient was singular, and *vice versa*, as in the sentence “chi legano gli orsi?,” *Who do the bears tie?*. The authors find that mismatch improves accuracy due to a transparent distinction between elements and the consequent lack of similarity effects.

Overall, the main finding across languages is a selective problem with both the subject and object *which* questions in acquisition, with several substitutions of the Wh- element and the emergence of strategies to avoid the full movement of the complex which NP element (see Belletti and Guasti, 2015 for comprehensive accounts; Thornton, 2016). What emerges from these studies is that children are following a trajectory in the acquisition of Wh- questions—and syntactic dependencies in general—starting from simpler subject questions (*who* subject) and then moving to more complex object extraction with NP restriction (*which* object). This trajectory should be considered in continuity with the development of the grammatical system and investigated within a set of predictions for the acquisition of each grammatical structure.

A model which fits the developmental stages in the acquisition of syntactic dependencies was recently proposed based on the notion of minimality between arguments (Friedmann et al., 2009). The model, originally developed to address the canonicity pattern reported in adults with language disorders (Garraffa and Grillo, 2008), suggested that young children have an immature grammatical system with poor production and comprehension of sentences with a moved object and intervening material, in particular when there is structural similarity between the two (Garraffa, 2017), as schematized in (1). According to the authors, children are more sensitive to effects of similarity between arguments, which leads them to adopt a restricted version of minimality: the model makes clear predictions that unlike adults, any representation like the one in (1a) and (1b) is equally perceived as a violation in children, with no full disjunction in the specification of the grammatical features of the arguments.

**Table:** 

(1)
1a. + A… + A… < + A > (identity)
–UNGRAMMATICAL
1b. + A, + B… + A… < + A, + B > (inclusion)
–UNGRAMMATICAL FOR CHILDREN
1c. + A… + B… < + A > (disjunction)
–GRAMMATICAL FOR CHILDREN

The generalization that emerges is that if the target of the movement and the intervening subject argument are sufficiently different in their internal featural composition, the configuration is unproblematic (e.g., a Wh- question with one animate and one inanimate argument). The defining factor appears to be the presence or absence of a lexical NP restriction. This model assumes that the source of difficulties in children’s grammatical development is based on a partial encoding of the grammatical information, not sufficient to parse sentences similar to (1b). Children therefore adhere to a stricter version of the locality principle, requiring distinct feature specifications for the target and for its intervener, and imposing a disjoint specification.

It is interesting to note that in these immature grammatical systems, an internal grammatical pressure of coping with the next level of the configuration can determine the production of sentences that are severely dispreferred in adults and not attested in the standard varieties. This is the case, for example, for children’s production of passives in Italian, which are substituted with a set of forms that are not attested in adult varieties (see Belletti, 2017 for details) or the case of the non-adult-like *which* questions with extra copies of the movement of the Wh- element in English (as in Thornton, 1995).

### Wh- questions among children with developmental language disorder

The comprehension and production of Wh- questions have been extensively investigated in typical language acquisition (Thornton, 2016). Research shows that the ability of children with DLD to produce interrogatives is not consistent with that of their age-matched peers (Levy and Friedmann, 2009). A few structural aspects of Wh- questions were examined in the research on DLD, including word order (canonical vs. non-canonical), the difference between moved Wh- and *in situ* Wh-, and the difference between Wh- argument and Wh- adjunct. The inclination of many researchers is to study comprehension of Wh- questions in terms of subject Wh- vs. object Wh- questions, exploring the canonicity pattern and the factors underlying the discrepancy in comprehension between the two structures (Ebbels and van der Lely, 2001; Deevy and Leonard, 2004; Friedmann and Novogrodsky, 2011).

Based on the word order of sentences, it was found that children with DLD found acquiring specific Wh- questions particularly challenging. Ebbels and van der Lely (2001) studied four English-speaking children with DLD (aged 11–13 years) that showed that even after a language intervention program, they were still unable to comprehend questions *which* (object) compared with the structure of Wh- subject questions *who*, *what*, and *which* (subject). Interestingly, studies by Wong et al. (2004) and Friedmann and Novogrodsky (2011) on Hebrew- and Mandarin-speaking children with DLD, respectively, supported this finding. This seemed to suggest that the structure of *which* (object) is a structure that is difficult across languages, an intuition which is theoretically supported by the concept of movement, as was proposed in some relevant accounts of the phenomenon. One such account is provided in the study by Friedmann and Novogrodsky (2011) who suggested that movement is responsible for the difficulties faced by children with DLD. The feature checking requirements of Wh- questions initiate the movement of an element (the Wh- element itself) crossing over the subject position to reach a higher position, as sketched in (1). This operation, which creates a dependency between the moved element and its trace that is interrupted by the subject, is understood to be difficult in children in intervention accounts.

The syntactic difficulty faced by children with DLD has been described, for example, by the deficit in computational grammatical complexity (DCGC) theory (Deevy and Leonard, 2004; Marinis and van der Lely, 2007). This theory presupposes that this difficulty is caused by the generation of highly complex sentences that involve movement utilizing various cycles of derivations (van der Lely and Stollwerck, 1997). The fact that object questions have been identified as complex is supported by studies such as van der Lely and Battell (2003) on English-speaking children, who found children with DLD to consider movement application as an optional phenomenon. In terms of produced structures, questions produced by children with DLD are not grammatical as verified by van der Lely and Battell’s (2003) study. Hamann’s (2006) study on Wh- questions among French-speaking children with DLD showed that these children did not produce Wh- fronting questions. Instead, they produced Wh- *in situ* questions. These studies describe an atypical development of Wh- dependencies, potentially due to an immature system. The pattern of produced sentences by children with DLD is of sentences that are more derivationally economical, avoiding any movement of elements on the left positions and echoing the order of the declarative sentence. Hamann’s (2006) findings were supported by Hansson and Nettelbladt’s (2006) findings who also found that Swedish-speaking children with DLD produced sentences that can be described as more economical due to the avoiding of fronting the Wh- element. Similarly, Jakubowicz (2011), eliciting different Wh- dependencies in French, shows that long-distance dependencies are avoided by both children with DLD and TD children, but children with DLD and younger TD children in particular resort to ungrammatical structures when a long Wh- dependency is elicited.

As discussed in this session, the structure of non-canonical Wh- questions derived through movement is a difficult structure for children with DLD, and a potential explanation for this difficulty is based on the presence of an intervener (the subject), which causes an effect of similarity between arguments. In fact, this difficulty toward which object structures is universal in nature as it is found across many languages.

### Wh- questions in Malay

Malay has both Wh- *in situ* questions and Wh- questions with movement (Kader, 1981; Salleh, 1989; Razak, 2003). The moved Wh- form is the grammatical form used in the standard Malay (SM) variety, particularly in the written form. In this variety, the Wh- word is fronted, and the interrogative affix -*kah* is attached to the questioned constituent, and the relative particle *yang* is present. The colloquial Malay (CM) variant has both the *in situ* and the moved Wh- forms, with the former being the most common. In the moved form, the interrogative affix -*kah* is absent, but the relative particle *yang* is present. Table 1 provides a full list of examples for each condition in the two varieties.

**TABLE 1:** Examples of Wh- interrogative sentences for standard Malay and colloquial Malay.

Structures	Standard Malay	Colloquial Malay	
	Fronted	Fronted	*In situ*
Who subject	Siapakah yang menangis? Who-PRT-Q that ACT-cry “Who was crying?”	Siapa yang menangis? Who that ACT-cry “Who cried?”	Siapa menangis? Who ACT-cry “Who cried?”
Who object	Siapakah yang kanak-kanak tarik? Who-PRT-Q that child pull “Who was the child pulling?”	Siapa yang kanak-kanak tarik? Who that child pull “Who did the child pull?”	Kanak-kanak tarik siapa? Child pull who “Who did the child pull?”
Which subject	Budak lelaki yang manakah menangis? Child male that which-PRT-Q ACT-cry “Which boy was crying?”	Budak lelaki yang mana menangis? Child male that which ACT-cry “Which boy cried?”	Budak lelaki mana menangis? Child boy which ACT-cry “Which boy cried?”
Which object	Budak manakah yang dia pilih? Child which-PRT-Q that he choose “Which boy did he choose?”	Budak mana yang dia pilih? Child which that he choose “Which boy did he choose?”	Dia pilih budak yang mana? He choose child that which “Which boy did he choose?”

Wh- *in situ* questions are questions in which the Wh- word constituent appears in the base position in the sentence, and the sentence conforms to the SVO word order. In the Wh- *in situ*, the Wh- word is in the base position and a raising intonation marks the sentence as an interrogative. In CM, for both *in situ* and moved options, the grammatical interrogative particle *kah* is not required. On the other hand, in SM, the moved Wh- questions comprise sentences that are generated from the base position and then undergo movement to the specifier position of the CP, and the specification of the relative particle *kah* on the Wh- element is obligatory (Razak, 2003). Affix *kah* is an overt morphological marker of interrogation in the specifier position, and it agrees with the particle *yang*. The *yang* construction in Malay is found in both SM and CM. Generally, it functions as a *yang-*type restrictive relative clause headed by *yang* (REL). Its function is to modify the head noun in a complex NP construction. Other functions of *yang* include *yang* as a deictic marker in a focused construction (“Yang tu kuat”/That one is strong) and *yang* as a complementizer with a [+ Q] feature (Malay also having *bahawa* [-Q], and the null complementizer [C ø]) (Wong, 2008). In Wh- questions as exemplified in Table 1, *yang* can be interpreted as a [+ Q] complementizer.

According to Wong (2008), in Wh- question formation, any argument in a position lower than that of a subject has to be passivized to become a derived subject before the extraction can occur. The Wh- phrase moves to the specifier position of an obligatory interrogative *yang*. The specifier, being an argument position, does not allow extraction from a position other than the highest subject position, a derived subject. Subjects in embedded or subordinate clauses can be questioned, provided the matrix verb is passivized as in (2) (example from Wong, 2008).

**Table:** 

(2)	Siapakah	yang	dikatakan (oleh)	
	Who-PRT-Q	that	PASS-say (by)
	John	akan	membeli	buku itu?
	John	will	ACT-buy	book the
	“*Who did John say will buy the book?*”			

In the context of the Malay language, studies on the ability of children with DLD with Wh- questions are limited compared with studies on Wh- questions of TD children (Aman, 2007, 2014; Kader and Tan, 2022). Studies on the language acquisition of TD Malay children confirm that there exist specific stages in the linguistic development of children. Long (1993) discovered that the majority of Malay children aged 5 and 6 years can understand and use verb and noun affixes in their school and home settings. Affixation is a pervasive morphological process in Malay and a prerequisite to produce standard Malay Wh- sentences. Importantly, an acquisitional trajectory was identified in the process, where older children master affixation more than younger children. In a longitudinal study of the spontaneous speech of five Malay children between the ages of 18 and 48 months collected weekly over a period of 2 years and 6 months (Tan, 1999; see Razak, 2013 for an overview), it was determined that the first Wh- words to appear are *mana*/where, *apa*/what, and *siapa*/who for children between 26 and 30 months. These are followed by *kenapa*/why (at 31 months) and *macam mana*/how (at 34 months). The last Wh- word to be acquired and rarely used is *berapa*/how much (35–36 months). Later, combined Wh- words such as *preposition* + *wh- word* and *wh- word* + *particle* appear, as in *dekat siapa*/near whom, *dengan siapa*/with whom, *dengan mana*/which one, *untuk apa*/for what, *macam ap*a/like what, *kat mana*/where at, and *macam mana*/how.

Another study collected naturalistic data from two Malay-speaking children around the age of 3 over a period of 3 months (Aman, 2007). The main finding of the study was the presence of both moved and *in situ* questions in the speech of both parents and children. However, an asymmetry between arguments and adjuncts was reported, with a preference for the *in situ* structure for the arguments in both parents and children. For particular adjunct questions (*how* and *why*), there was a strong tendency to select the moved question structure. This asymmetry between *in situ* arguments and moved adjuncts was reported in both short and long questions. A proposal to explain the asymmetry in Malay children is that it relies on *in situ*, rather than displaced, constructions to produce questions as a strategy to avoid any non-local dependencies (Cole and Hermon, 1998). A follow-up step in their grammatical development will be to attach all obligatory elements to the verb, thus licensing Wh- elements in the left periphery of the clause.

According to the model proposed by Aman (2007), TD children acquiring Malay will first make use of the *in situ* strategy and subsequently acquire a new grammatical operation. This operation is the generation of a gap without the need to reconstruct the Wh- element. It is possible that acquiring this mechanism is hard for children with DLD, who will prefer to stick with a simpler available version, compatible with CM. If this is the case, namely, if children with DLD do not fully acquire gap constructs for Wh- questions, then they will struggle to understand and produce certain Wh- questions, particularly NP restricted Wh- questions (e.g., *Siapakah yang membaca buku itu*? Who reads the book?) due to the necessary specification of an operator in the CP domain. In this account, TD children will go on to acquire more complex operations that they will be able help in the comprehension and production of Wh- constructions.

An interesting matter to explore is the reason behind this lack of progress in the grammatical development of children with DLD, assuming similar language exposure between DLD and TD children and the impact of the educational system on the grammar. The standard variant of Malay is part of the curriculum taught in primary schools, including the introduction of more complex sentences such as focused questions, as in *Buku yang Mary beli* (“It was a book that Mary bought”).

### Current study

The present study aims to expand on previous findings on the acquisition of grammar in Malay children with DLD looking at syntactic abilities in both comprehension and production of Wh- questions. It investigates the abilities of children on different Wh- questions, aiming to explore whether there are differences among them and to record the strategies in place to overcome more complex structures.

## Materials and methods

### Participants

The study sample comprised three groups of children speakers of Malay as a dominant language and attending public government schools in Malaysia: one experimental group and two control groups. The DLD group comprised 15 children with a diagnosis of DLD (12 boys and three girls; age range 7;0–9;11 years); the control group matched by chronological age (CA) comprised 15 children (12 boys and three girls) with typical language; and the second control group was matched by language abilities (LA) and consisted of 15 children (age range 4;0–6;11 years). CA participants were matched by age to DLD on a one-to-one basis (± 2 months), and LA participants were matched on performance on a linguistic assessment. Subjects from both control groups had normal hearing, as reported by their parents.

The 15 children with DLD were recruited from a pool of students who obtained C, D, and E grades in their Malay language subject in the year-end school examinations. They failed the national LINUS examination, which screens students in year 1 for the 3Rs—reading, writing, and arithmetic in addition to reasoning, and were placed in remedial classes (Luyee et al., 2015). There was an initial total of 26 subjects recruited; however, four students did not meet the normal score of Raven’s Colored Matrices Test, and seven students failed to obtain consent from their parents/caregivers to participate in this study. All children were clinically diagnosed with language impairment and were receiving treatment at the time of testing. Their status was confirmed using a battery of baseline tasks that assessed the children’s non-verbal and verbal abilities. Raven’s Colored Progressive Matrices Test (Raven et al., 1998) was used to measure the children’s non-verbal abilities, which were within the norm. The subjects were screened by an audiologist, and they had normal hearing (not exceeding 25 dB), and from an SLT through an oro-motor assessment that determined there were no articulatory conditions interfering with language. The screening for language included the Malay Preschool Language Assessment Tool (MPLAT, Razak et al., 2018), the sentence repetition task, and school grades in the Malay language subject. The MPLAT assessment is a standardized tool that has normative data of 510 Malay children aged 4;0–6;11 years. It tests both receptive and expressive language and early literacy skills of Malay preschool children. Table 2 is a summary of the linguistics components included in the MPLAT.

**TABLE 2:** Linguistics components included in the MPLAT screening test (Razak et al., 2018).

Dimension	Modality	Task
Morphology	Comprehension	Picture vocabulary Sentence comprehension
Syntax	Repetition	Sentence repetition
Semantics	Comprehension/production	Referential meaning Relational meaning
Early literacy skills	Reading/writing	Awareness of alphabets, alphabet-sound correspondence, copying, spelling skills

Results from the MPLAT were used to determine the language-matched group. Children with DLD obtained a score of −2 SD below the average standard score for their age-group, and they were thus matched with children belonging to the age-group whose scores were similar to those of the participants with DLD, as shown in Table 3. A *t*-test for independent sample confirmed there is no significant difference between the children with DLD and the language-matched control group (*p* = 1.81). These results showed that the communicative ability of children with DLD lies within the ability range of preschool children (LA group).

**TABLE 3:** Demographic details and language scores on the MPLAT components and school grades for Malay language for the three groups.

	DLD (SD)	Age-matched (SD)	Language–matched (SD)
Age (SD)	9;7 (1.79)	9;67 (1.79)	5;73 (1.75)
**MPLAT scores**			
**MPLAT overall scores**	72.53 (11.60)	100.00 (0.00)	83.69 (29.4)
MPLAT receptive language	45.3 (7.5)	100.00 (0.00)	50.2 (14.5)
MPLAT expressive language	27.2 (8.05)	100.00 (0.00)	33.49 (19.4)
**School language scores**			
Grammar score (School grade)	29.43 (13.93)	78.21 (8.61)	61.7 (16.78)
Composition score (School grade)	26 (9.08)	71.36 (6.41)	NA

### Task materials

A total of two tasks were adapted to Malay in order to assess comprehension and production of Wh- questions, one from Friedmann and Novogrodsky (2011) and one from Jakubowicz (2011).

### Sentence comprehension

Sentence comprehension was explored with a sentence to picture matching task, targeting arguments in subject and object positions. The task was composed of 40 items: 10 *Siapa/who* subject questions, 10 *yang mana/which one* subject questions, 10 *Siapa/who* object questions, and 10 *yang mana/which one* object questions. Examples of the four structures are provided in (3)–(6).

**Table:** 

(3)	*Siapa* in subject:		
	Siapa	cium	adik?
	Who	kiss	little sister/brother
	“Who kissed little sister/brother?”		

**Table:** 

(4)	*Yang mana* in subject:		
	Nenek	yang mana cium	adik?
	Grandmother which	kiss	little sister/brother
	“Which grandmother kissed little sister/brother?”		

**Table:** 

(5)	*Siapa* in object:			
	Siapa	yang	adik	cium?
	Who	that	little sister/brother	kiss
	“Who did little sister/brother kiss?”			

**Table:** 

(6)	*Yang mana* in object:			
	Nenek	yang mana	adik	cium?
	Grandmother	which	little sister/brother	kiss
	“Which grandmother that little sister/brother kissed?”			

**FIGURE 1:**
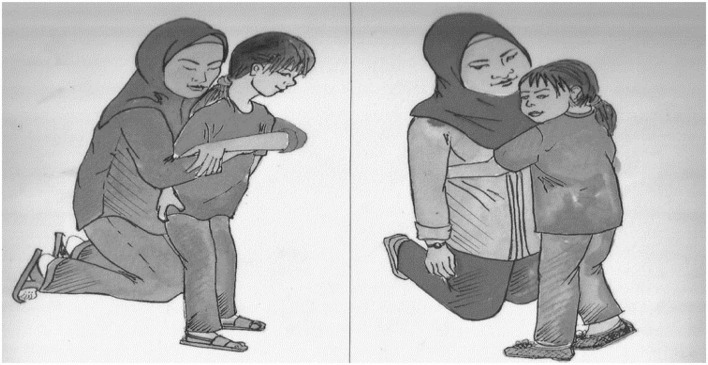
Picture pair used for the sentence “*Emak mana yang adik peluk*?” (*Which mother is little daughter hugging?*).

All items in the comprehension tasks were moved Wh- sentences in line with the grammar of SM, but with the absence of the -*kah* interrogative particle. In the sentence–picture matching task, the children listened to the recorded sentence and were asked to point to the two pictures that matched the sentence. The stimuli for the tasks included a picture set and an audio recording (see Figure 1).

### Elicitation of Wh- questions

In this task, children are instructed to ask questions to a puppet with the appearance of a cartoon character. A total of 40 items were provided to prompt the child to ask Wh- questions with four different configurations, namely, *who subject* (10 items), *who object* (10 items), *which subject* (10 items), and *which object* (10 objects) questions.

To elicit the production of a Wh- element, part of the picture stimulus was hidden. The child is instructed to ask the puppet “Angry Bird” about the hidden information, as exemplified in (7) for *Siapa*/who subject question.

(7) Elicitation of a *Siapa*/who subject question:

Preamble: Itik sedang makan. Kita tak tahu nama orang yang beri itik makan. Cuba adik tanya *angry bird*.

*The duck is eating. We do not know the name of the person who is feeding the duck. Please ask Angry Bird who.*

Expected answers:

**Table:** 

(7a)	Moved Wh:				
	Siapa	yang	beri	itik	makan?
	*Who*	*that*	*give*	*duck*	*eat*
	“*Who is feeding the duck?*”				

**Table:** 

(7b)	*In situ* Wh:				
	Yang	beri	itik	makan	siapa
	*That*	*give*	*duck*	*eat*	*who*
	“*Who is that (person) who gave the duck food?*”				

**FIGURE 2:**
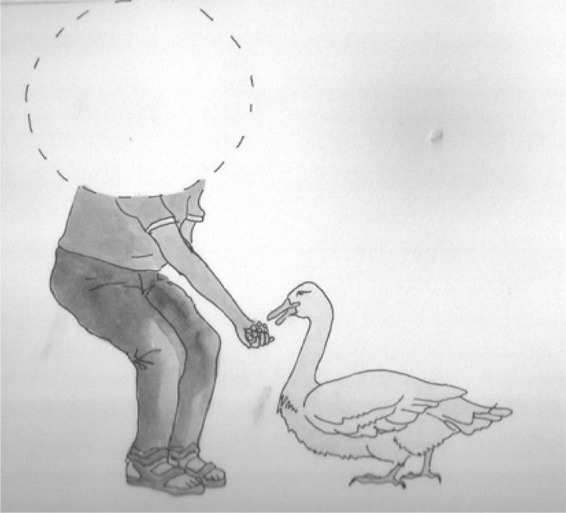
An example for elicitation of the Wh- sentences *Siapa yang beri itik makan?* (*Who is feading the duck?)*.

An example of the elicitation material is offered in Figure 2.

### Scoring

The scoring procedures followed the scoring method used by Aman (2007). In the comprehension task, a score of 1 was given if the children’s answers matched the target pictures and a score of 0 if the children’s answers did not match the target pictures. For the production task, a score of 1 was given if the children’s responses matched the situations given. Substitution of nouns/personal pronouns (e.g., *ibu/mother* is replaced by *kakak/older sister*), use of contracted forms (e.g., *tidak/*NEG to *tak*), and deletion of the open syllable (determiner *ini/this* to *ni*) were still considered correct if the structure of the question matched the elicited question. A score of 0 was given if the children’s answers resulted in a change in the original structure to another syntactic structure, sentences that change the target sentence’s meaning. Because elicited contexts were felicitous with both a moved Wh- element and an *in situ* construction, all felicitous answers were further analyzed for the type of answer provided. These were “movement” and “*in situ.*”

In terms of the qualitative analysis, errors committed by children in the production task were transcribed and divided into structural and lexical errors. Structural errors encoded errors in the omission of Wh- questions, order of sentences that differed from the target sentences, incorrect usage of the Wh- elements, and ungrammatical sentences. Lexical errors encoded errors in the addition, omission, or substitution of lexical items.

### Reliability

A second speech and language therapist native speaker of Malay transcribed productions from two children. The reliability of the transcription was measured by using a formula that calculates the percentage agreement for verbatim transcriptions. The results showed that the reliability between assessors was around 93%. Scoring reliability was also enforced using the test–retest method on five children from the entire subject population.

## Results

Table 4 presents accuracy results across tasks (comprehension and production). Inferential statistics were run on R Studio (R Studio Team, 2022) and Jamovi (The Jamovi Project, 2021) and repeated measures ANOVA were implemented.

**TABLE 4:** Comprehension and production overall performance across the three groups.

Group	Comprehension %	Production %
DLD	85.5	46
Age-matched	99.2	98.3
Language-matched	87.2	50.7

### Comprehension

Table 5 presents accuracy in the comprehension of the four Wh- questions tested (who subject/who object and which subject/which object) in the three groups.

**TABLE 5:** Comprehension of Wh- questions (standard deviation) for the three groups.

Sentence type	DLD (*n* = 15)	Age-matched (*n* = 15)	Language-matched (*n* = 15)
*Siapa* (Who subject)	94 (0.88)	100 (0.00)	96.67 (1.02)
*Yang mana* (Which subject)	90 (0.89)	99.3 (0.25)	94 (1.09)
*Siapa* (Who object)	88 (1.09)	99.3 (0.25)	90 (1.26)
*Yang mana* (Which object)	70 (1.98)	97.3 (0.57)	74 (1.96)

A repeated measures ANOVA was run to compare the effect of group and condition (who subject, who object, which subject, which object). There was a significant difference in score between groups [*F*_(2,42)_ = 17.1, *p* < 0.001], a significant difference between conditions [*F*_(3,103)_ = 26.61, *p* < 0.001], and a significant interaction between groups and conditions [*F*_(6, 103)_ = 4.77, *p* < 0.001]. Tukey’s *post-hoc* comparisons revealed there was a significant difference between the group of children with DLD and age-matched controls (*p* < 0.001) but not between children with DLD and language-matched group (*p* = 0.78). The only operator to be significantly different from all others is *yang mana* (which object) (*p* < 0.001). In terms of the interactions between groups and conditions, *post-hoc* comparisons reveal that children with DLD are significantly worse than age-matched controls only in the *yang mana* (which object) condition (*p* < 0.001) and not on all others, while they are not statistically different from the language-matched group in any of the conditions.

### Production

A repeated measures ANOVA was run to determine whether there was a significant effect of group and condition (viz., type of operator: why/who/where/what) on accuracy in production. There was a significant effect of group [*F*_(2, 42)_ = 26.4, *p* < 0.001], but not of condition [*F*_(3, 126)_ = 1.71, *p* = 0.16], or their interaction [*F*_(6, 126)_ = 1.61, *p* = 0.15]. Tukey’s *post-hoc* comparisons run for group determined that significant differences appear between the DLD group and the age-matched group (*p* < 0.001), but not the language-matched group (*p* = 0.82).

A second analysis was run on the target answers produced in the elicitation task to check whether group determined differences in the type of answer selected across all conditions, namely, *in situ* or movement. As described in the introduction, both options are grammatical in CM, although only movement structures are grammatical in SM.

Table 6 shows results across all Wh- elements in the three groups. A repeated measures ANOVA was run to determine the effects of group on the type of answer selected. The results of the ANOVA show a significant effect of the interaction between groups and types of answer [*F*_(2, 1984)_ = 82.2, *p* < 0.001]. Tukey’s *post-hoc* comparisons revealed that children with DLD are significantly different from CA both in the selection of *in situ* (*p* < 0.001), which is selected 1.7% of the time by CA and 27% of the time by DLD, and movement (*p* < 0.001), which is selected 96% of the time by CA and 18% of the time by DLD. No differences are reported between children with DLD and LA children.

**TABLE 6:** Answer types provided across all conditions in Wh- question elicitation for the three groups.

Sentence type	DLD (*n* = 15)	Age-matched (*n* = 15)	Language-matched (*n* = 15)
*In situ*	27 (6.6)	1.7 (0.94)	25 (7.63)
Movement	18 (6.19)	96.5 (1.4)	27.5 (9.93)
Non-target	54.3 (8.34)	1.8 (1.06)	47.3 (12.12)

## Error analysis

Children’s errors in producing the utterances were analyzed and are reported in Table 7. Errors committed by the subjects were grouped into two categories, namely, structural and lexical errors. The total number of errors committed by children with DLD and the language-matched group is comparable for all sentence structures. The two groups also share the main error types, that is, substitution with echo questions (declaratives with interrogative intonation) and wrong use of Wh- elements, whereas this type is not reported in the age-matched group.

**TABLE 7:** Types and occurrences of structural errors in Wh- question productions for the three groups.

Types of errors	DLD	Age-matched	Language-matched
Substitution with Eco Questions	17	1	11
Wrong use of Wh-	9	1	15
Incorrect Wh- movement	2	0	0
Verb omission	3	0	2
Insertion of *yang*	1	0	2

Children with DLD and language-matched children tended to substitute Wh- questions with echo questions, declarative sentences with no Wh- element, and the insertion of the NP. An example of substitution with an echo question is reported in (8).

**Table:** 

(8)	Target sentence				
	Abang	makan	nasi	kat	mana?
	Brother	eat	rice	at	where
	“Where did brother eat rice?”				

**Table:** 

	Subject’s Response				
	Abang	makan	nasi	kat	dapur?
	Brother	eat	rice	at	kitchen
	“Brother ate rice in the kitchen?
	” (DLD SB: K, 5;2).				

The second most frequent error produced by both language-matched children and children with DLD was the use of the wrong Wh- word, considering the context given and the expected targeted Wh- word and adopting a generic mana (“where”) element.

**Table:** 

(9)	Target response				
	Kat	mana ayah	pasang	khemah?
	At	where	father	set up	tent
	“Where did father set up the tent?”				

**Table:** 

	Subject’s Response				
	Kenapa	ayah	pasang	khemah?
	Why	father	set up	tent
	“Why father set up the tent?
	” (DLD: MI, 8;7).				

A second error classification on lexical errors is proposed in Table 8. These are errors are not apparently targeting a grammatical property and mainly targeting the knowledge of the verbs. It is interesting to note that the age-matched group did not produce any lexical error, showing a fully-fledged mastery of the verbal domain.

**TABLE 8:** Lexical errors across the structure of Wh- questions.

Types of errors	*In situ*			Movement		
	DLD	Age-matched	Language-matched	DLD	Age-matched	Language-matched
Verb substitution	7	0	0	1	0	2
Preposition substitution	2	0	1	3	0	8
Lexical additions	1	0	2	0	0	2

The most frequent type of lexical errors was the substitution of verbs with another semantically related form. An example of a lexical error of verb substitution is seen in (10). The targeted *belajar*, “to study” was substituted with the verb *mengajar*, “to teach.”

**Table:** 

(10)	Target response			
	Abang	belajar	dengan	siapa?
	Elder	brother	study	with whom
	“*With whom did elder brother study?*”			

**Table:** 

	Subject’s response			
	Abang	mengajar	dengan	siapa?
	Big	brother	Aff-teach	with who
	“*Who did elder brother teach?*”
	(DLD: 8:8 years old).			

### Malay Wh- questions as clinical markers

To explore the sensitivity and specificity for Wh- questions, a comparison between the performance of children with DLD and their peers, the CA group, was conducted. A value of 2 standard deviations below the mean value of the CA group was used as suggested in the literature (Conti-Ramsden et al., 2001; Paul et al., 2012). The calculation of the sensitivity and specificity values used 80% as minimum value and 90% and above as good/excellent for clinical markers (see Bortolini et al., 2006 for more information). A set of proposed clinical markers for Wh- questions, defined as the elements that could characterized the DLD profile, are presented in Table 9 in terms of the sensitivity, specificity, and accuracy values for difficult Wh- structures for Malay children with DLD.

**TABLE 9:** Sensitivity, specificity, and accuracy values for Wh- structures.

Sentence structure	Sensitivity	Specificity	Accuracy
Comprehension *siapa*/ Who (subject)	53% (8/15)	100% (15/15)	77% (23/30)
Comprehension *yang mana*/Which one (subject)	67% (10/15)	87% (13/15)	77% (23/30)
Comprehension *yang mana/* Which one (object)	93% (14/15)	100% (15/15)	7% (29/30)
Production *-wh*	87% (13/15)	93% (14/15)	90% (27/30)

The results showed that for the comprehension of Wh- sentences, the Wh-*yang mana*/which one (object), has the best sensitivity value (93%) and excellent specificity value (100%). This supports the finding that Wh- sentences with *yang mana/which* one are the most difficult for children with DLD and a potential candidate to be investigated in further studies.

## Discussion

The study reported a set of data on Wh- sentence production and comprehension in a group of children native speakers of Malay. In this article, two main findings have been reported when comparing the performance of typical children and children with DLD, namely, a selective deficit for comprehension of which questions and a clear asymmetry in the production for children with DLD.

Where they produced *in situ* questions, age-matched children prefer to move the Wh- element at the root of the sentences and create a filler–gap relation as required in standard Malay. More interesting from a developmental point of view is the convergence of results between children with DLD and the younger group of language-matched children, making a strong case for a delay in the language development of DLD compatible with a pre-stage of language development. Malay younger children and children with DLD seemed to adhere to a similar timetable, and they have not developed structures that can be described as dependent or late acquired, for example, long-distance dependencies with lexical restricted items.

**FIGURE 3:**
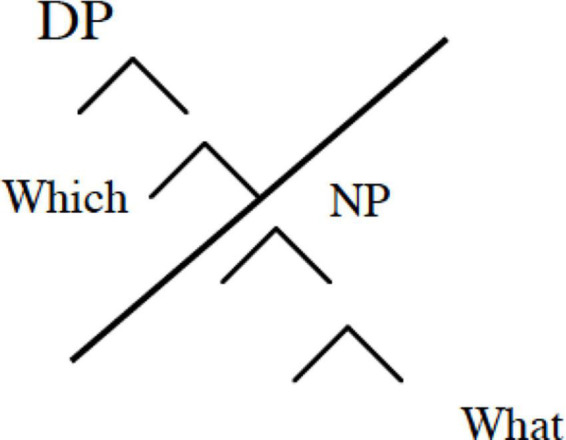
A truncated structure for acquisition of Wh- questions.

From a theoretical point of view, the data on interrogatives discussed in this article can be interpreted as an instance of grammatical reduction of the formal features necessary to activate the upper part of the syntactic tree. The outcome of this specific reduction could be a structure at play in both younger children and children with DLD, truncated in Rizzi’s sense as shown in Figure 3 (Rizzi, 1993/1994). This reduced structure allows the activation of the left periphery of the clause with base-generated placeholders, but it is not rich enough to license the movement on which NP restricted elements, favoring a lower *in situ* position for these elements.

The results are evidence collected from the literature for operators, such as which NPs, and need to be licensed as DPs to be permitted in the upper part of the syntactic tree. This can be formally represented, for example, as pied-piper features that have to be included in the derivation of the structure during its numeration and not later (see Watanabe, 2006 for a more detailed explanation). If the pied-piper features are not placed as a result, for example, of an underrepresented structure, the computation cannot proceed further, and effect like minimality but based on a minimal logical form (LF) representation can be the cause of an underspecified/reduced representation. In the case of children acquiring Malay, it is possible that a principle like ‘‘avoid pronoun’’ or a similar principle of structural economy is a play with the effect of minimal pied piping at LF representation, placing the reduction of the syntactic tree at the interface between syntax and semantic. This is also supported by the qualitative analyses of the errors with a preference for a selection of generic operators, echo questions, and, in general, a selective impairment of critical features to allow which-X operator to be represented as DPs^1^.

Overall, the results of our research show an asymmetry between comprehension and production in both atypical and early typical language acquisition. However, a detailed analysis of the linguistic strategies adopted to carry out the production task, which allows to generate alternatives to express information in a way that is more in line with the person’s grammatical knowledge, shows differences in the language systems of the participants. All the strategies adopted aim at avoiding the more complex syntactic computation involved in long syntactic dependencies of more complex operators. Crucially, resorting to this tactic means that the syntactic structure present in SM is not a strong option in the language systems of DLD and young children, as exemplified by the difficulty found in the production of any Wh- moved question. We would like to argue that the data reported support the idea that the movement strategy, while present to some extent, is not mastered in both children with DLD and younger kids. An asymmetry was found where both control children and participants with DLD comprehend all question types with movement, suggesting that they have acquired the computation involved in movement of restricted elements. However, a difference emerges between conditions, where one type of moved question is selectively impaired, namely, which object questions. Because movement as an operation is present in the abstract representations of these children, the issue with this specific occurrence of it is finer grained. A suggested interpretation will be given shortly.

If comprehension of Wh- questions with movement is generally mastered, younger controls and participants with DLD struggle with their production. In fact, when asked to produce questions, both groups produce non-target answers about half of the time, and they do not show a preference for either *in situ* of moved structures when they produce target answers, regardless of the elicited operator (which/who/where/when), in clear contrast with older control children who overwhelmingly prefer the production of moved structures. This point is crucial to underline the importance of testing children in more than one modality.

Considering all these pieces of evidence, the discrepancy between comprehension and production in a parallel testing ground is a fruitful method for evaluating grammatical knowledge. Where linguistic development is grammatically consistent, linear heuristics are not adopted, and implementation of a syntactic algorithm is preferred to the use of a “good enough” extralinguistic strategy. Findings indicate that both younger children and children with DLD face difficulties in production compared with comprehension, as previously reported for other languages (Contemori and Garraffa, 2010). But a modality explanation does not cover the more detailed pattern of errors visible in production, with children with DLD facing a delay in the acquisition of discourse-linked questions. This result is consistent with the findings of Ebbels and van der Lely (2001) and Friedmann and Novogrodsky (2011), supporting the idea of a delay in the acquisition of selective instances of Wh- movement. This matter can be proven for the more complex extractions of *which* questions as in *kakak yang mana* which functions as object in (12).

**Table:** 

(12)	[kakak yang mana]_i_	adik	kejar	t_i_?
	↑_______________________________|			

There are two options to explain the selective deficit with which object questions for this group: it can be described as a consequence of a reduction of the featural representation, leading to more intervention errors due to the lexical restrictions on which questions, or of a more structural reduction related to a truncated structure (as in Figure 3), where the edge of the syntactic tree is omitted in younger children and children with DLD. The production data in the present study support the second model, showing the relative absence of movement of Wh- elements to the left periphery in DLD and younger children but not older children, who learn it as a by-product of education. It seems to be the case that in the case of poor language learners such as individuals with DLD, these are not able to move toward the next step of the syntactic structure. This statement was supported by Aman’s (2007) study who reported an effect of exposure to the variation of a standard language on children’s syntactic abilities.

With regard to sentence production, the results of this study show that a significant difference occurs between the performance of the children with DLD and the age-matched group. Children with DLD have acquired and prefer the Wh- *in situ* structure compared with TD children of their same age, who use moved structures of different kinds, for example, Wh- fronting with subject–auxiliary inversion. In the context of the Malay language, it is possible that the use of productive Wh- movement questions among age-matched children has a connection with their exposure to a formal learning of grammar that is the prescribed grammar of standard Malay in schools. Both groups of children aged 7–9 years received exposure to the Malay language, which follows the rules set by Karim et al. (2009:15), a prescribed grammar book that provides an explanation of Malay grammar and is used as reference grammar by schoolteachers. In standard Malay grammar rules, the Wh- movement question is the only option allowed for questions. It is possible that exposure through formal learning has an influence on the differences between the two subject groups.

This study also examined aspects of errors committed by the three subject groups. Results show that there is an inclination for children with DLD and language-matched children to omit the Wh- element and produce an Echo question, namely, a declarative question with an interrogative intonation. The omission of Wh- elements is not surprising in the language acquisition process (Gerken, 1994; Schmerse et al., 2013). An interesting error reported in our DLD group is the substitution of the Wh- element with a generic *mana* (where). Such a strategy shows that children produce Wh- questions, but they express them in an arbitrary manner without considering the context of the sentence and, more importantly, making use of an element that does not require any interference with the subject position. This assumption is consistent with results reported in Long’s (1993) study for affixation, with Malay children using affix forms arbitrarily during acquisition, following a grammatical underspecification strategy. The use of a generic Wh- placeholder is also supported by the pervasive error of verb omission reported in children with DLD in this study, who tend to produce phrasal utterances instead. According to Aman (2007), the omission of verbs is one of the sentence simplification strategies adopted by young children speakers of Malay.

Regarding lexical level errors, this study findings reveal that children tend to substitute verbs with other verbs with same interference in the semantic relation, for example, an antonymous relationship (push for pull). A similar error was recently reported in the interpretation of active reversible sentences in a group of Malay speakers with aphasia (Aziz et al., 2020) with lexical substitutions in favor of semantically related verbs. These results were explained as an underspecification of the grammatical affixes in transitive verb forms (e.g., agentive markers and voice), often reported and theorized in adults with acquired language impairments as well as in children with DLD (Garraffa and Grillo, 2008; Adani et al., 2010).

## Clinical implications

Examining sentence structures that might potentially be a clinical marker for Malay children with DLD, the threshold score of the CA group was used as the cutoff point to measure sensitivity and specificity for all constructions, in support of a syntactic structure-based approach to clinical markers. This was implemented as there were significant differences on the performance of children with DLD compared with the TD Malay children of the same chronological age. The results obtained in this study strongly suggest that Which-questions, and in particular the comprehension of Which-object questions, are possible candidates to be linguistic clinical markers in Malay. Future studies are required to further corroborate the results on a larger population and to further investigate acquisition of *which* questions in children with DLD. A follow-up study making use of a syntactic priming paradigm specific for *which* questions could better explore whether children with DLD can acquire any Wh- dependencies under a controlled setting and with more exposure to the structure (see Garraffa et al., 2018 for a study on the acquisition of relative clauses in DLD *via* syntactic priming).

## Conclusion

This study represents a major contribution to the investigation of language development in children speakers of Malay and provides finer details on information regarding the ability and language development of children with DLD. Overall, the study reveals that Malay children with DLD at this stage (mean age 9;7 years) master comprehension of most Wh- questions, but not production, thus confirming a modality-driven component, which has been reported in several other studies for both TD children and children with DLD. However, in terms of the description of syntactic abilities of children with DLD, a modality-driven approach cannot explain the variation of both structural and lexical errors reported in the atypical group, as well as the selective difficulty with which object questions. In terms of quality, an analysis of errors shows that although quantitatively similar, the language make-up of children with DLD has some differences with that of younger, age-matched children.

One aspect of the late acquisition of Wh- questions in children with DLD is linked to the extraction of the Wh- from its argument position, supporting studies on Wh- questions across languages which show difficulties in understanding which questions. In the case of Malay as reported here, children with DLD adopt a series of strategies that appear to be related to an immature or truncated syntactic tree. This reduced tree allows for non-adult-like optional constructions, including Wh- *in situ* questions, use of a generic Wh- element, substitution with yes/no echo questions, and, at lexical level, incorrect use of verbs.

Factors such as the formal education of Malay were reported to have an influence on the usage pattern of Wh- questions in the older children, but not for children with DLD. Children with DLD at the ages of 7–9 years are still unable to use the particle *–kah* compared with age-matched children. The implications of formal education on the acquisition of grammatical properties and the need for extra support for the children with atypical language development need to be explored further.

## Data availability statement

The original contributions presented in this study are included in the article/supplementary material, further inquiries can be directed to the corresponding author.

## Ethics statement

The studies involving human participants were reviewed and approved by the Research Ethics Committee, Universiti Sains Malaysia (USM). Written informed consent to participate in this study was provided by the participants’ legal guardian/next of kin.

## Author contributions

NA conceptualized the research, collected and analyzed the data, drafted the work for intellectual content, gave final approval of the version to be published, and was responsible for the accuracy and integrity of data and that the data have been investigated and resolved thoroughly. GS and MG analyzed the data, drafted the work for intellectual content, gave final approval of the version to be published, and was responsible for the accuracy and integrity of data and that the data have been investigated and resolved thoroughly. RR conceptualized the research, analyzed the data, drafted the work for intellectual content, gave final approval of the version to be published, and was responsible for the accuracy and integrity of data and that the data have been investigated and resolved thoroughly. All authors contributed to the article and approved the submitted version.

## Footnotes

1 In natural languages interesting linguistic evidence related to the syntax-semantic interface have been presented in support of a positional distinction of Wh operators based on semantic properties of definiteness restriction (Heim, 1987). In her investigation on Wh-traces and definite variables Heim presents data related to “definiteness” of the moved wh-phrase, (example below). (11) a. ?? Which one of the two men was there in the room/*drunk? b. ?? Which actors were there in the room/*laughing? c. ? Who was there in the room when you got home? d. What is there in Austin? A sort of definiteness hierarchy seems related to the possibility of extraction of a Wh operator in “there is” constructions, with “What” the less specified operator and a licit extractable element. This fact could allow us to describe the non-standard linguistic competence of our children, with all extractions are treated as the case in d, the one with the less specified operator.
